# Corticosteroid Treatment in Diabetic Macular Edema

**DOI:** 10.4274/tjo.56338

**Published:** 2017-06-01

**Authors:** Burcu Nurözler Tabakcı, Nurten Ünlü

**Affiliations:** 1 Ağrı State Hospital, Ophthalmology Clinic, Ağrı, Turkey; 2 Ankara Training and Research Hospital, Ophthalmology Clinic, Ankara, Turkey

**Keywords:** Diabetic macular edema, intravitreal corticosteroid, triamcinolone acetonide, dexamethasone, fluocinolone acetonide

## Abstract

Diabetic macular edema is the most common cause of visual impairment in patients with diabetes mellitus. The pathogenesis of macular edema is complex and multifactorial. For many years, laser photocoagulation has been considered the standard therapy for the treatment of diabetic macular edema; however, few patients achieve significant improvements in visual acuity. Today the intravitreal administration of anti-inflammatory or anti-angiogenic agents together with the use of laser photocoagulation represents the standard of care for the treatment of this complication. The intravitreal route of administration minimizes the systemic side effects of corticosteroids. Steroid-related ocular side effects are elevated intraocular pressure and cataract, while injection-related complications include endophthalmitis, vitreous hemorrhage, and retinal detachment. In order to reduce the risks and complications, intravitreal implants have been developed recently to provide sustained release of corticosteroids and reduce repeated injections for the management of diabetic macular edema. In this review, the efficacy, safety, and therapeutic potential of intravitreal corticosteroids in diabetic macular edema are discussed with a review of recent literature.

## INTRODUCTION

Diabetic macular edema (DME) is the leading cause of vision loss in patients with diabetic retinopathy (DR). In the WESDR (Wisconsin Epidemiologic Study of Diabetic Retinopathy), the 10-year incidence of DME was 20.1% among patients with type 1 diabetes, 13.9% among type 2 diabetics using insulin, and 25.4% among type 2 diabetes patients not using insulin.^1^ Without timely and appropriate treatment, DME leads to permanent vision loss. Although the rate of serious vision loss due to DME is believed to have fallen in recent years, an additional 12,000-24,000 new cases are reported each year.^2^

Grid and focal laser photocoagulation have long been accepted as the standard treatment for vision loss associated with DME. It has been shown that laser photocoagulation reduces the risk of moderate vision loss in DME; however, many patients are unable to regain lost vision and the procedure is not effective in all DME patients.^3^

With the development of intravitreal agents such as anti-vascular endothelial growth factor (anti-VEGF) and steroids, new strategies are now recommended for the management of this complex disease. While intravitreal implantation offers potential visual gains compared to laser interventions, repeated application confers risks in terms of both drug- and surgery-related side effects.^3^ With the longer duration of effect provided by intravitreal implants, the aim is to provide better visual recovery and fewer side effects. This review discusses the pathogenesis of DME, the rationale behind the use of corticosteroids, and current approaches to steroid use in the management of DME.

### Pathogenesis of Diabetic Macular Edema

The pathogenesis of DME is complex and multifactorial. DME forms as a result of fluid accumulation in the retinal layers due to disruption of the blood-retina barrier (BRB). Hyperglycemia is the main risk factor for DR. Hyperglycemia causes high intracellular glucose levels, free radical production due to oxidative stress, and activation of protein kinase C. Chronic hyperglycemia leads to the formation of advanced glycation end products. Advanced glycation end products in the vitreous and vitreoretinal interface are responsible for the neurovascular damage seen in DR.^4^

The nervous and vascular systems are parallel systems in embryonic development. The two systems support each other during the formation of the vascular and nerve structures. Microvascular leakage and neuronal apoptosis occur in a mutual interaction. In the retinal neurovascular unit, Müller cells act as a bridge between the retinal nerves and the microcirculation. Müller cells are also an important component in the BRB. Cytoplasmic swelling in Müller cells is an early sign of macular edema, resulting in the accumulation of extracellular fluid within the cells.^5^

Other causes such as hypoxia, impaired blood flow, retinal ischemia, and inflammation are also associated with the progression of DME. Elevated VEGF levels, endothelial dysfunction, leukocyte adhesion, reduced levels of pigment epithelium-derived factor, and increased protein kinase C production lead to BRB destruction and increased vascular permability.^4^

VEGF is a homodimeric glycoprotein which stimulates vascular endothelial cell proliferation and increases vascular permeability. VEGF-A stimulates microvascular leakage and neuronal apoptosis, and is critical in the neurovascular unit.^5^ Many studies have shown that VEGF plays an important role in the development of DME.^6,7^ The main anti-VEGF agents used in the treatment of DME are ranibizumab, bevacizumab, pegaptanib sodium, and aflibercept.^3^

Although the contribution of VEGF to the development of DME is indisputable, the role of other non-VEGF pathways has also been emphasized. There are many studies demonstrating the role of inflammation in the development of DR. Research on steroids in the treatment of DME has been ongoing for many years due to their powerful antiinflammatory and antiedematous effects. Corticosteroids block the arachidonic acid pathway via phospholipase A2 inhibition. This inhibits the synthesis of thromboxanes, leukotrienes, and prostaglandins, and prevents vasodilation and increased capillary permeability. Corticosteroids also stabilize lysozymes, reduce synthesis of inflammatory mediators and VEGF, inhibit cell proliferation, stabilize the BRB, enhance the density and activity of tight junctions in the retinal capillary endothelium, and improve retinal oxygenation.^8^

Significant decreases in retinal thickness have been observed within 1 hour of intravitreal triamcinolone acetonide (IVTA) injection, though no change was seen with bevacizumab after 24 hours.^9^ Steroids are fast-acting due to non-genomic interactions with the plasma membranes, independent of gene transcription. The inhibition of osmotic swelling in Müller cells due to endogenic adenosine release also contributes to the rapid improvement in retinal thickness after IVTA injection. Endogenic adenosine release activates A1 receptors and opens glial potassium and chloride channels. The outflow of ions stabilizes the osmotic gradient and prevents cellular swelling. IVTA also stabilizes Starling forces by reducing vasoconstriction and hydrostatic pressure.^8^

Anti-VEGF agents are the first choice in pharmacologic treatment of DME. However, 61% of the patients in the RISE-RIDE study did not show visual gains of 15 letters or more, and 43% did not achieve visual acuities of 20/40 or better. The limited visual gains in those patients was believed to be related to neural damage, retinal pigment epithelium changes, and subretinal fibrosis resulting from chronic macular edema (mean duration, 4.5 years) prior to treatment, as well as structural damage from repeated macular laser therapy, and the natural course of DR.^10^

Nonresponse to anti-VEGF therapy can be defined as a lack of anatomic improvement or the recurrence of retinal exudation when the interval between injections is extended. Steroid therapy should be considered in such cases.

Intravitreal steroid injections reduce DME and stabilize vision, but side effects are common. The most common side effects are elevated intraocular pressure (IOP) and cataract formation. Therefore, steroids are preferable in pseudophakic eyes that have persistent or recurrent disease. Steroid therapy for DME is administered as peribulbar injection, intravitreal injection, or intravitreal implant. There are currently three different intravitreal steroids utilized: triamcinolone acetonide, fluocinolone acetonide, and dexamethasone.

### Triamcinolone Acetonide

TA is a synthetic steroid with five times the anti-inflammatory strength of hydrocortisone. TA has a long-acting profile due to its low water solubility. The therapeutic effect of intravitreal 4 mg TA persists for up to 3 months.

IVTA in suspension form is currently available as the following commercial preparations: Trivaris (Allergan, Irvine, CA, USA), Kenacort (Bristol-Myers-Squibb, Melbourne, Australia) and Kenalog (Bristol-Myers-Squibb, Princeton, NY, USA).^3^ IVTA was first utilized in the treatment of age-related macular degeneration, and within a few years began to be used in the treatment of DME as well.^11,12^ The sub-Tenon route was initially preferred for steroid injections to treat DME, but it was later established that intravitreal injection was more effective in treating refractory DME.

A prospective study conducted by Diabetic Retinopathy Clinical Research Network (DRCR.net) compared the safety and efficacy in the 3-year results of preservative-free 1 mg and 4 mg IVTA versus focal/grid laser therapy. Patients were randomly assigned to one of 3 groups receiving either focal/grid laser treatment, 1 mg IVTA, or 4 mg IVTA. After 4 months of treatment, the group receiving 4 mg IVTA showed the largest gains in best corrected visual acuity (BCVA), but there was no significant difference in BCVA between the groups at 1 year. At 2 years, mean BCVA was highest in the laser group, which was confirmed by central retinal thickness (CRT) measurements taken with optical coherence tomography. At 3 years, the laser group showed a BCVA increase of 5 letters, while neither IVTA group showed a change in BCVA from baseline. In terms of side effects observed in the laser, 1 mg IVTA, and 4 mg IVTA groups, IOP elevation over 10 mmHg occured in 4%, 18%, and 33%, and the probability of cataract surgery increased by 31%, 46%, and 83%, respectively. Therefore, it was concluded that IVTA did not provide long-term benefits in the treatment of DME compared to laser photocoagulation.^13,14^

Following the publication of the DRCR.net study demonstrating that laser therapy was superior to IVTA, a phase 2b clinical trial of a triamcinolone sustained delivery intravitreal implant (I-vation, Surmodics, Inc., MN, USA) was terminated.

DRCR.net later initiated a large randomized clinical study comparing laser photocoagulation with two different intravitreal agents in the treatment of central DME. Patients were randomly divided into 4 groups: sham injection + prompt laser, 0.5 mg intravitreal ranibizumab (IVR) + prompt laser, 0.5 mg IVR + deferred laser, and 4 mg IVTA + prompt laser. At 1 year, improvements in BCVA were significantly greater in the IVR + prompt laser and IVR + deferred laser groups when compared with the IVTA and laser-only groups. Compared to the laser-only group, all 3 of the groups that received intravitreal injections showed significant and comparable decreases in CRT.^15^ The results of extended follow-up at 2 years were consistent with those published at 1 year.^16^ Mean changes in BCVA compared to the laser-only group were +3.7 letters in the IVR + prompt laser group, +5.8 letters in the IVR + deferred laser group, and -1.5 letters in the IVTA + prompt laser group. Visual improvement in phakic eyes receiving IVTA was limited by the incidence of cataract. Cataract surgery was necessary in 55% of patients receiving IVTA, compared to 12% in the IVR group. Among the pseudophakic eyes in that study, BCVA outcomes were better in the IVTA + prompt laser group compared to the laser-only group, and were comparable to those in the IVR groups. However, the risk of IOP elevation was higher in the IVTA group (38%) than in the IVR + prompt laser group (5%). It was concluded that IVR injection is effective in DME, and that IVTA is an alternative option for pseudophakic eyes.

Currently, the intravitreal application of triamcinolone acetonide to treat DME is an off-label use. For this reason, IVTA is recommended either alone or in combination with laser therapy in selected patients with persistent and refractory DME and vision loss, particularly pseudophakic patients.^3^

### Dexamethasone

The intravitreal dexamethasone implant (DEX implant; Ozurdex, Allergan) contains 0.7 mg preservative-free dexamethasone, can be stored at room temperature, and is applied using a pre-loaded 22-gauge intravitreal injector system. It is a biodegradable, sustained-release implant which remains effective for up to 6 months.

The MEAD study was a 3-year, randomized, sham-controlled study evaluating the safety and efficacy of the 0.35 mg and 0.7 mg intravitreal DEX implants in DME. The mean number of injections over 3 years was 4.1, 4.4, and 3.3 in the 0.7 mg DEX implant, 0.35 mg DEX implant, and sham injection groups, respectively. At the end of follow-up, a visual gain of ≥15 letters was achieved in 22.2% of the patients that received 0.7 mg DEX implant, 18.4% with 0.35 mg DEX implant, and 12% in the sham injection group. The largest reduction in mean central macular thickness was observed in the 0.7 mg DEX implant group (-111.6 µm), followed by the 0.35 mg DEX implant group (107.9 µm) and the sham group (-41.9 µm). In terms of adverse effects, the rate of cataract development among phakic patients was 67.9%, 64.2%, and 20.4% and IOP increases >10 mmHg occured in 27.7%, 24.8%, and 9.1% in the 0.7 mg DEX implant, 0.35 mg DEX implant, and sham injection groups, respectively. IOP elevation was controlled in most cases with or without medication, but trabeculectomy was necessary for 2 patients (0.6%) in the 0.7 mg DEX implant group and 1 patient (0.3%) in the 0.35 mg DEX implant group.^17^

In the PLACID study, patients with diffuse DME randomly received either 0.7 mg DEX implant or sham injection, both followed by laser photocoagulation after 1 month. When necessary, a second DEX implant or sham injection was given 6 months after the initial injection, and in both groups up to 3 supplemental laser applications were done at 3-month intervals. The DEX implant and laser group showed a greater decrease in vascular leakage and retinal edema on angiography compared to the group treated with laser only. There was no significant differences between the groups in BCVA at 12 months. However, BCVA was significantly increased in the DEX implant group at 1 and 9 months. IOP elevation over 10 mmHg occured in 15.2% of patients in the DEX implant group, but was controlled without the need for glaucoma surgery. At 12 months, 3.2% of the patients had undergone cataract surgery.^18^

The BEVORDEX study was a randomized clinical study comparing bevacizumab and 0.7 mg DEX implant in patients with DME. The study included 88 eyes of 61 patients with central DME. Forty-two eyes received pro re nata intravitreal bevacizumab every 4 weeks, and 46 eyes received a pro re nata DEX implant injection every 16 weeks. BCVA increases of 10 letters or more were observed in 40% of eyes treated with bevacizumab and 41% of eyes treated with DEX implant. None of the eyes that received bevacizumab showed BCVA decreases of 10 letters or more, while 11% of the eyes that received DEX implant had vision loss, mostly due to cataract. Central macular thickness decreased by a mean of 122 µm in the bevacizumab group and 187 µm in the DEX implant group. Mean number of injections over 12 months was 8.6 among eyes treated with bevacizumab and 2.7 among eyes that received DEX implant.^19^

The CHAMPLAIN study reported the 26-week outcomes of 55 vitrectomized patients with refractory DME lasting for a mean of 43 months who were treated with 0.7 mg DEX implant. Mean change in CRT from baseline (403 µm) was -156 µm at 8 weeks and -39 µm at 26 weeks. Mean increase in BCVA was 6.0 letters at 8 weeks and 3.0 letters and 26 weeks; at 8 weeks, 30.4% of patients had increases of 10 letters or more and 42.9% of patients had increases of 5 letters or more. The study demonstrated that in vitrectomized eyes with refractory DME, the DEX implant had an acceptable safety profile and provided statistically and clinically significant visual gains as well as reduced vascular leakage.^20^

Overall, the risk/benefit ratio of the DEX implant is favorable in pseudophakic patients or a limited patient group who do not respond to nonsteroid therapies or for whom these therapies are not suitable.

### Fluocinolone Acetonide

Intravitreal fluocinolone acetonide (IVFA) is commercially available in two different extended-release drug delivery systems, Retisert (Bausch&Lomb, Rochester, NY, USA) and Iluvien (Alimera Sciences, Atlanta, GA, USA).

Retisert is a nonbiodegradable implant containing 0.59 mg fluocinolone acetonide. It is implanted through a pars plana incision and sutured to the sclera and continuously releases the drug for up to 30 months. After surgical implantation, it initially releases the steroid at 0.6 µg/day, which gradually decreases over the first month and stabilizes at approximately 0.3-0.4 µg/day.^21^ Retisert has been approved for the treatment of noninfectious posterior uveitis.

In a multicenter study investigating the safety and efficacy of Retisert in the treatment of persistent and recurrent DME, patients were randomly assigned to receive either 0.59 mg IVFA or observation/additional laser photocoagulation (standard of care). BCVA increased by 3 or more lines in 16.8% of the IVFA-implanted eyes at 6 months, 16.4% at 1 year, 31.8% at 2 years, and 31.1% at 3 years, compared to 1.4%, 8.1%, 9.35%, and 20% at the same time points in the eyes that received standard care. Throughout the study, implanted eyes showed greater reductions in CRT when compared to the standard care group. By the end of a 4-year follow-up period, 91% of the phakic eyes treated with IVFA required cataract surgery. IOP elevation ≥30 mmHg was observed in 61.4% of IVFA-implanted eyes, and 33.8% underwent a surgical procedure to control IOP.^22^

Iluvien is a nonbiodegradable implant containing 250 µg fluocinolone acetonide. It is injected into the vitreous using a 25 G injector and releases 0.5 or 0.2 µg/day of active agent. Three-year follow-up outcomes have been published for the multicenter, double-blind FAME study about the efficacy of Iluvien implant in patients with DME refractory to laser therapy. Patients with DME who had received laser therapy at least once were randomly assigned to 3 groups: 0.2 µg/day IVFA (low dose), 0.5 µg/day IVFA (high dose), or sham injection. Visual gains of 15 letters or more were reported in 28.7%, 27.8%, and 18.9%, respectively, at 3 years. Treatment was repeated at 12 months in 25% of the patients. Patients were able to receive laser treatment 6 months after initial treatment, and 40% of the patients underwent additional laser therapy. In addition, subgroup analysis was conducted to investigate the effect of DME duration on treatment. Among chronic patients with DME duration of 3 years or more, BCVA gains of ≥15 letters were observed in 34% (low dose), 28.8% (high dose), and 13.4% (sham); the improvements in the steroid implant groups were significantly greater. However, this difference was not significant among patients with DME for less than 3 years. Although all of the patients treated with IVFA developed cataract, their visual gains after cataract surgery were comparable to those of pseudophakic patients. After 3 years, incisional glaucoma surgery was required in 4.8% of the patients in the low dose group and 8.1% in the high dose group.^23^

Intravitreal administration of corticosteroids reduces their systemic side effects and confers several advantages in the treatment of DME. Because anti-VEGF agents are administered at frequent intervals, treatment costs are high. Sustained-release steroid implants reduce the number of intravitreal injections and greatly lower the risk of endophthalmitis and traumatic cataract. However, the risk of developing corticosteroid-induced cataract is extremely high.

Although there is better patient compliance with the IVFA implant because its duration of effect is longer than the DEX implant, but it has also been associated with higher risk of ocular hypertension and cataract. However, no clinical studies directly comparing the two treatment methods have been conducted to date. There is insufficient evidence that repeated administration of a DEX implant does not carry the same risks as sustained-release IVFA.

In brief, anti-VEGF agents are recommended as initial treatment for DME involving the central macula. Laser photocoagulation is preferable in patients with noncentral DME due to the low risk, low cost, and patient compliance in this group. There are no studies comparing anti-VEGF agents and sustained-release corticosteroid therapy in the initial treatment of central DME.

Steroid therapy allows suppression of both the inflammation and the VEGF pathway in DME. The duration of effect is longer, and the injection number and follow-up frequency are lower than with anti-VEGF treatment. Therefore, particularly in chronic diffuse macular edema, steroid therapy is preferable for patients who do not respond to anti-VEGF therapy, or who have conditions contraindicated for anti-VEGF therapy such as recent cerebrovascular event or myocardial infarction. Due to the short half-life and probable low efficacy of anti-VEGF agents, steroid implants may be appropriate as initial treatment in vitrectomized patients with central DME. Corticosteroid implants are suitable alternatives to anti-VEGF therapy for pseudophakic patients with persistent central DME who do not have significant risk of glaucoma.^24^

In addition to monotherapies, the long duration of effect of corticosteroid implants may enable combination therapies. However, clinical studies are still needed to evaluate the synergistic effects of these implants used in combination with laser and anti-VEGF agents.
